# Online peer support in rheumatology: a mixed methods exploration of patient and clinician views and experiences

**DOI:** 10.1093/rheumatology/keag492

**Published:** 2026-09-11

**Authors:** Shihab Ahmed, Martha Piper, Michael Bosley, Shaista Tayabali, Alice Tunks, Sean Humfrey, Wendy Diment, Max Yates, James A Bourgeois, Melanie Sloan

**Affiliations:** School of Clinical Medicine, University of Cambridge, Cambridge, UK; Department of Public Health and Primary Care Unit, University of Cambridge, Cambridge, UK; Patient and Public Partner; Patient and Public Partner; Department of Public Health and Primary Care Unit, University of Cambridge, Cambridge, UK; Patient and Public Partner; Patient and Public Partner; Faculty of Medicine and Health Sciences, University of East Anglia, Norwich, UK; Department of Psychiatry and Behavioral Sciences, University of California, Davis Medical Center, Sacramento, CA, USA; Department of Public Health and Primary Care Unit, University of Cambridge, Cambridge, UK; Faculty of Medicine and Health Sciences, University of East Anglia, Norwich, UK

**Keywords:** patient experience, mixed methods, primary care rheumatology, mental health services, social support, patient attitude to health, attitude of health professionals, SLE, RA, Sjogren’s syndrome

## Abstract

**Objectives:**

Online peer support groups (OSGs) are increasingly used by patients with rheumatic diseases, yet their benefits, risks and impact on the clinician–patient relationship are not well characterized. We examined how patients and clinicians perceive and experience OSGs.

**Methods:**

Mixed methods combining online surveys and interviews with rheumatology patients and clinicians. Measures of satisfaction with life and care were compared by *t*-tests between OSG members and non-members. Free-text and interview data underwent thematic analysis.

**Results:**

Three themes were identified from surveys (*n* = 1316 patients, *n* = 317 clinicians) and interviews (*n* = 19 patients, *n* = 14 clinicians). (1) Validation and affirmation: OSGs provided emotional reassurance, understanding and belonging, especially those with rare or ‘invisible’ diseases who felt disbelieved by others. (2) Patient empowerment through health literacy: OSGs filled informational and relational gaps left by time-limited healthcare systems, offering practical, experience-based advice. Although 85% of patients reported finding helpful information on OSGs, 48% reported misinformation circulating in groups. Effective moderation was viewed as key. (3) Tensions and inequities within peer spaces: 71% of clinicians viewed OSGs as helpful, while many worried about negativity and distrust in healthcare systems. Members described ‘toxic positivity’, ‘toxic negativity’ and exclusionary dynamics within the OSGs, with younger, male and racially minoritized patients more likely to report feeling unwelcome.

**Conclusion:**

OSGs can offer meaningful emotional and practical benefits to patients with rheumatic diseases, particularly those who feel marginalized in social or clinical contexts. However, these benefits coexist with challenges around misinformation, inclusivity and the clinician–patient relationship.

*Rheumatology* key messages

- Online peer support addresses emotional and informational needs unmet by healthcare systems.
- Patient empowerment coexists with risks including misinformation, burnout and strained patient–clinician relationships.
- Clinicians should engage constructively with peer support to promote safe, inclusive patient communities.

## Introduction

Patients with systemic autoimmune rheumatic diseases (SARDs) often experience a substantial and multifaceted disease burden, characterized by unpredictable and persistent symptoms such as chronic pain, fatigue and depressed mood [1, 2]. Consequently, these patients demonstrate a lower health-related quality of life (HRQoL) compared with the general population [3, 4]. Although advances in pharmacological management have improved disease outcomes, the emotional and informational needs of patients remain only partly addressed within conventional healthcare systems. Social support can improve wellbeing in chronic illness [5], yet access may be restricted by reduced mobility, stigma or geographical distance [6].

Peer support has increasingly gained recognition as a complementary form of care in chronic illness. Thompson *et al.* [7] define peer support as ‘a socially driven intervention involving people with lived experience of a condition helping others to manage the same condition’. Evidence from patients with cancer and cutaneous autoimmune conditions suggests that peer support can improve patient outcomes through shared experience, improved outlook and mutual understanding [8, 9]. Furthermore, research indicates that unmet informational needs drive patients, especially those with rare diseases, towards peer support programmes, which bridge gaps in practical and disease-specific knowledge [10].

Peer support has increasingly moved into digital spaces, especially since the COVID-19 pandemic. Online peer support groups (OSGs) are commonly organized on social media platforms, through charities such as Lupus UK. Research suggests that using online platforms enhances accessibility and extends engagement to those previously unable to participate in person [11]. However, evidence for measurable changes in patient outcomes remains limited; a review of online peer support networks in chronic illness found positive subjective experiences but inconsistent evidence of objective improvement of health outcomes [12].

OSGs may be particularly valuable in rheumatology, improving accessibility and bringing psychological and informational benefits. Yet the overall impact of peer support in this context remains uncertain, as interventions vary widely in design, definitions of ‘peers’ and the types of support offered [7, 13]. Existing literature offers limited clarity regarding which elements of peer support are most effective for patients with rheumatic diseases, and how such initiatives might be optimized [14]. Despite the rapid expansion of online peer support, little is known about how these platforms are perceived and utilized by either patients or clinicians. Understanding both perspectives is essential, as online peer interactions may influence clinical relationships, self-management behaviours and patient wellbeing.

This study aimed to explore the experiences and perceptions of online peer support among patients with SARDs and clinicians. By integrating patient and clinician perspectives, this work seeks to identify the perceived benefits, risks and implications of online peer support for rheumatology practice.

## Methods

### Design

Quantitative data were collected through two surveys: one for patients with rheumatic diseases, another for clinicians involved in the care of such patients. Qualitative data were collected through free-text survey responses and semi-structured interviews of patients and clinicians, asking their views on OSGs.

### Patient involvement

Patients and clinicians, from a variety of backgrounds, were involved in every stage of this study.

### Materials

Patients completed Likert-scale survey questions assessing adaptation, coping and satisfaction with medical care, alongside the Warwick–Edinburgh Mental Wellbeing Scale [15]. A free-text section captured additional comments. Clinicians were asked about their views on OSGs in a wider clinician survey on healthcare-seeking behaviours, and in interviews encompassing questions on healthcare-behaviours and medical relationships.

We followed recent guidelines on producing quality surveys [16]. During the design process, understanding of survey questions was tested with patients and clinicians, including using the ‘think aloud’ method [17].

Semi-structured interviews were carried out with patients and clinicians. Interviews were conducted with guiding questions about the participants’ perceptions of peer support and its effects on them as a patient or in their clinical practice, with appropriate probing from the interviewers. Interview content was guided by relevant recent literature, alongside patient and clinician input. The audio content of the interviews was recorded and transcribed. Interviews continued until thematic saturation.

Most patient interviews were carried out by online video conferencing or telephone by MS and MU, both experienced female interviewers, one White, one South Asian. A minority of patients opted to receive questions by email (*n* = 5). Clinician interviews were conducted by MS. Interview schedules encompassed a range of topics about living with SARDs and patient–clinician interactions.

### Participants

Patients were recruited in 2021 via social media, professional networks and OSGs. The surveys were completed online, using Qualtrics, by patients who were 18 years or older and self-declared their autoimmune disease. Patients selected their disease from a list or responded with free text for ‘other’ diseases, later verified to be rheumatic diseases. Patients and clinicians who agreed to be interviewed were purposively sampled to ensure a range of socio-demographics characteristics, diseases (patients) and specialties (clinicians). Due to the limited number of minoritized ethnicity participants, additional interviewees were sought from a related study into experiences of participants from minoritized ethnicities [18].

### Ethics

Ethical approval was given by the Cambridge Psychological Society: PRE.2019.099 and PRE.2020.089. All participants provided informed consent either through the online surveys on Qualtrics or verbally before interviews. All data were handled in accordance with the Data Protection Act, 2018 [19].

### Data analysis

The ‘triangulation protocol’ was used to integrate quantitative and qualitative data which involved consideration of where the types of data agree, disagree or complement each other [20, 21].

Quantitative data were analysed descriptively using SPSS (IBM, Armonk, New York), including *t*-tests, ANOVA and bi-variate correlation analyses. A pre-agreed statistics plan was used, which involved selecting variables of interest and analysis methods. Statistical significance was *P* = 0.05.

For qualitative analysis, additional quotes were taken from related interviews conducted by team members and from the open-ended survey questions on peer support. These were not included in the number of interviews. All qualitative data were imported into NVivo, from where thematic analysis was carried out, as outlined by Braun and Clarke, involving immersion, coding and theme refinement as a team [22]. Quotes were selected illustratively to support themes, labelled ‘CS’ for the patient survey.

### Reflexivity

All team members reflected on how their own biases influenced the collection and interpretation of the data, and how the interview questions were constructed to minimize risk of bias. For example, alternative interpretations of participant responses were discussed during team meetings before themes were finalized.

## Results

Of the patient participants (survey, *n* = 1316, interviewees, *n* = 19), most were female (survey, 95%, interviews 89%). Three hundred seventeen clinicians responded to our survey and the majority were female (70%). Patient interviewees included 47% from minoritized ethnicities. Clinician interviewees (*n* = 14) included rheumatologists (43%) and GPs (21%) and were mostly male (57%) (Table 1).

**Table 1:** Participant characteristics.

	Characteristic	Patient survey		Patient interviews		Clinician survey		Clinician interviews	
		*n* = 1316	%	*n* = 19	%	*n* = 317	%	*n* = 14	%
Age	18–30	72	5.5	1	5.2	–	–	0	0
	30–39	149	11.3	4	21.1	–	–	4	28.6
	40–49	242	18.4	7	36.8	–	–	3	21.4
	50–59	386	29.3	3	15.8	–	–	4	28.6
	60–69 (60+ for clns)	315	23.9	3	15.8	–	–	3	21.4
	70+	152	11.6	1	5.3	–	–	–	–
	Prefer not to say		0.0	0	0	–	–	0	0
Gender	Female	1252	95.1	17	89.5	222	70.0	6	42.9
	Male	58	4.4	2	10.5	95	30.0	8	57.1
	Other/undisclosed	6	0.5	0	0	0	0	0	0
Member of OSG	Yes	1099	11.6	–	–	n/a	n/a	n/a	n/a
	No	217	16.5	–	–	n/a	n/a	n/a	n/a
Disease	SLE	412	31.3	6	31.6	n/a	n/a	n/a	n/a
	Inflammatory arthritis	416	31.6	2	10.5	n/a	n/a	n/a	n/a
	Vasculitis	47	3.6	3	15.8	n/a	n/a	n/a	n/a
	Sjögrens	116	8.8	2	10.5	n/a	n/a	n/a	n/a
	PMR	43	3.3	2	10.5	n/a	n/a	n/a	n/a
	UCTD	45	3.4	2	10.5	n/a	n/a	n/a	n/a
	Myositis	45	3.4	1	5.3	n/a	n/a	n/a	n/a
	SSc	100	7.6	0	0	n/a	n/a	n/a	n/a
	Mixed/multiple	88	6.7	1	5.3	n/a	n/a	n/a	n/a
	Other	1	0.1	0	0	n/a	n/a	n/a	n/a
Ethnicity	Asian	–	–	5	26.3	–	–	–	–
	Black	–	–	3	15.8	–	–	–	–
	Latino/a/x	–	–	1	5.3	–	–	–	–
	White	–	–	10	52.6	–	–	–	–
	Mixed	–	–	0	0	–	–	–	–
	Other	–	–	0	0	–	–	–	–
Specialty	Rheumatology	n/a	n/a	n/a	n/a	191	60.3	8	57.1
	GP/primary care	n/a	n/a	n/a	n/a	41	12.9	3	21.4
	Neurology	n/a	n/a	n/a	n/a	–	–	2	14.3
	Psychiatry	n/a	n/a	n/a	n/a	9	2.8	1	7.1
	Other	n/a	n/a	n/a	n/a	76	24.0	0	0

### Theme 1: Validation and affirmation: ‘A relief to know other people experience the same things as you.’ (CS1341, multiple diagnoses)

Members of OSGs described how they benefit from existential validation that affirms both the reality of symptoms and the validity of emotional responses to chronic illness. For many participants, the challenge of coping with chronic illness and multiple symptoms was exacerbated by a sense of isolation. Within peer support spaces, members found the space to share their own experiences. Subsequently, they described finding powerful reassurance and psychological relief.

*‘*[Fellow OSG members] *reassure me that I’m not “mental”* [and] *it’s not all in my head because many people talk about similar symptoms and experiences that I have’* (CS1636, SLE).

Many participants referred to ‘*feeling understood*’ (multiple patients) as a key benefit of OSGs. Patients reported that OSGs felt like a safe space to express emotions, to be vulnerable, honest and form deep connections.

Fellow patients offered empathy, authenticity and an emotional connection rooted not in training, but in illness experience, filling a relational void that formal healthcare professionals and those without SARDs do not reach.

‘*People understand the pain you suffer. They understand how it makes you feel*’ (CS1571, RA).

Most (62%) survey respondents reported that helping others in OSGs made them feel good about themselves (Fig. 1A).

**Figure 1:**
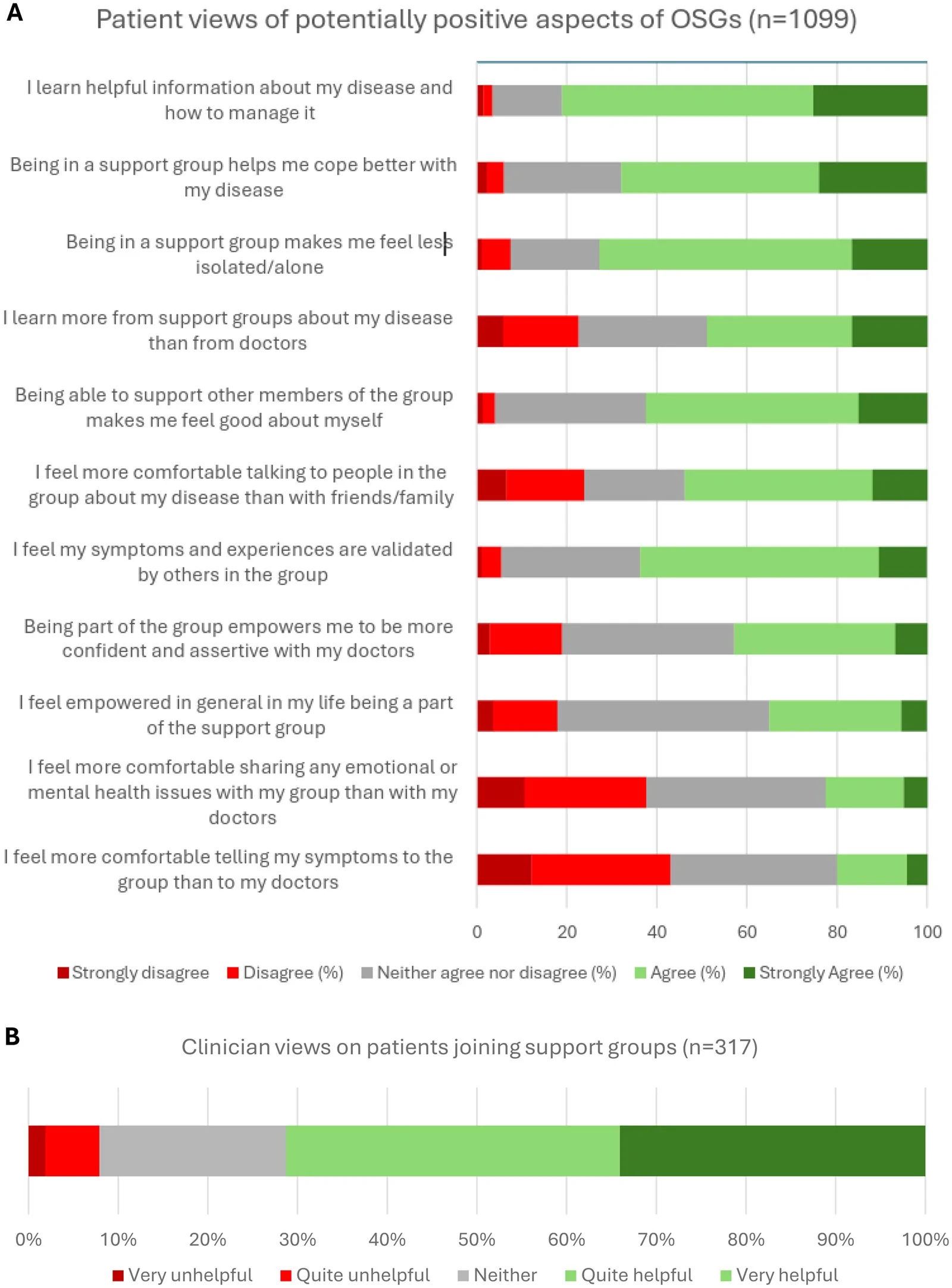
Views of OSG. (**A**) Patient views of positive aspects of OSGs. (**B**) Clinician views on level of helpfulness of OSGs

*‘I do like that I can do my best to reassure or support newly diagnosed “lupies” [patients with SLE] as I remember only too well how frightening it was 15 years ago for me*’ (CS1735, multiple diagnoses).

#### Subtheme: Isolation related to illness motivates patients to join OSGs

Some patients described experiencing ‘*isolation wrought by reduced mobility*/*social contact’* (CS0136, RA) and 73% of survey respondents stated that OSGs made them feel less alone (Fig. 1A).

*‘I am not alone in fighting this disease’* (CS0011, multiple diagnoses).

Peer support appears to be particularly important for patients living with rare and/or poorly understood conditions. ‘*Invisible conditions*’ (multiple patients) with less visible symptoms can result in reduced understanding and empathy from wider society:

*‘I find it hard to get support from friends and family as these symptoms are invisible and I feel that my friends/family cannot fully appreciate how ill I am as I look well*’ (CS0675, MCTD).

Paradoxically, the disease process puts vulnerable patients in greater need of support yet often makes that support harder to access (e.g. due to impaired mobility).

*‘With rare conditions there is not a lot of information readily available, so being part of an online community gives me the opportunity to ask questions about living with my disease’* (CS0237, SSc).

Patients and clinicians described how the support from OSGs contrasted with that of family and friends who sometimes encouraged them to ‘*tough it out and get more exercise and sunshine’* (Ppt 13, SLE) and clinicians, who were described by some patients as ‘*poo-pooing your symptoms’* (CS0681, SCLE).

### Theme 2: Patient empowerment and health literacy *‘*You learn a lot and learn to question more’ (CS1686, RA)

Participants often turned to peer groups for matters they considered less relevant to clinicians, such as managing side effects, diet or fatigue. OSGs serve as a reservoir of everyday strategies passed between patients, often invisible to the clinical gaze:

*‘One of the things I did was I shared my tips online, in terms of how to deal with [problems] when it comes to cooking, like opening up a jar*’ (Ppt 14, RA).

Patients spoke about the role of online support groups in providing tangible, daily utility. They describe how OSGs provided lay infrastructures of care, filling the informational and temporal gaps left by stretched healthcare systems:

*‘New patients diagnosed by GPs often feel particularly adrift because they don’t get the explanations they need to learn to live with their illness. In fact, day-to-day coping information is more likely to come from a support group’* (CS0939, Vasculitis).

Patients described how the asynchronous nature of chronic illness care means that distress can accumulate without timely medical reassurance. OSGs act as informal triage at all h and usually offer quicker responses.

*‘If I post on the forum, it’s usually because I’m between appointments and won’t be seeing my specialist for a while, and I just want a bit of human reassurance’* (CS0558, Vasculitis).

Furthermore, advice grounded in real-world experience was often seen as more actionable or relevant than clinician guidance, especially when healthcare systems were experienced negatively:

*‘I have learnt more about Sjögren’s from people with Sjögren’s than I have from most doctors that I have seen’* (CS0044, Sjögren’s).

OSGs thus help cultivate a form of experiential health literacy grounded in shared trial-and-error among patients, rather than top-down guidance from clinicians.

However, other patients preferred to take information from external sources, suggesting that OSGs ought to primarily signpost to scientific information, via clinicians or online research.

About 85% of survey participants agreed that they found helpful information about the disease and its management on OSGs (Fig. 1A).

*‘*[Peers] *keep up to date with emerging research’* (CS0203, RA).

#### Subtheme: Health literacy leading to agency and resistance

Patients used OSGs to become more confident in managing their illness and take a more active role in their care. Although clinicians reported encouraging patients to self-manage their symptoms, many acknowledged that: ‘*We just don’t have the time in clinics* [to assist with self-management]’ (Nurse 14).

Patients described how peers offered medical advice relating to medication dosage, changing therapies and managing side effects.

*‘When I was first prescribed* [cortico]*steroids I was given no help, either with long-term usage or side-effects. The forum members were the only ones who helped me’* (CS0502, Vasculitis).

Peer support functions not only emotionally but as a parallel form of care, shaping patient agency and preferences for help from those with lived experience.

*‘*[With time, peer support] *helps a person to feel more confident about having to battle symptoms of a chronic illness on daily basis*’ (CS0884, RA).

Patients described reclaiming expertise over their bodies and challenging medical hierarchies, demonstrating the role of OSGs as incubators of agency and resistance. Patients discussed empowerment as learning how to articulate their needs more effectively and gaining ‘*confidence to ask the right question’* (CS1561, Sjögren’s). Patients reported learning to request medications, challenge doctors or self-advocate for referrals.

*‘I’ve appreciated the knowledge and moral support when I felt I needed to stand up to my consultant and not be “railroaded” into* [taking cortico-] *steroid-sparing medication*’ (CS1131, multiple diagnoses).

Despite the reported empowerment, members of OSGs had significant lower levels of feeling in control (*P* < 0.001), yet non-significant higher levels of agreeing that they ‘know a lot about their disease’ (Fig. 2A).

**Figure 2:**
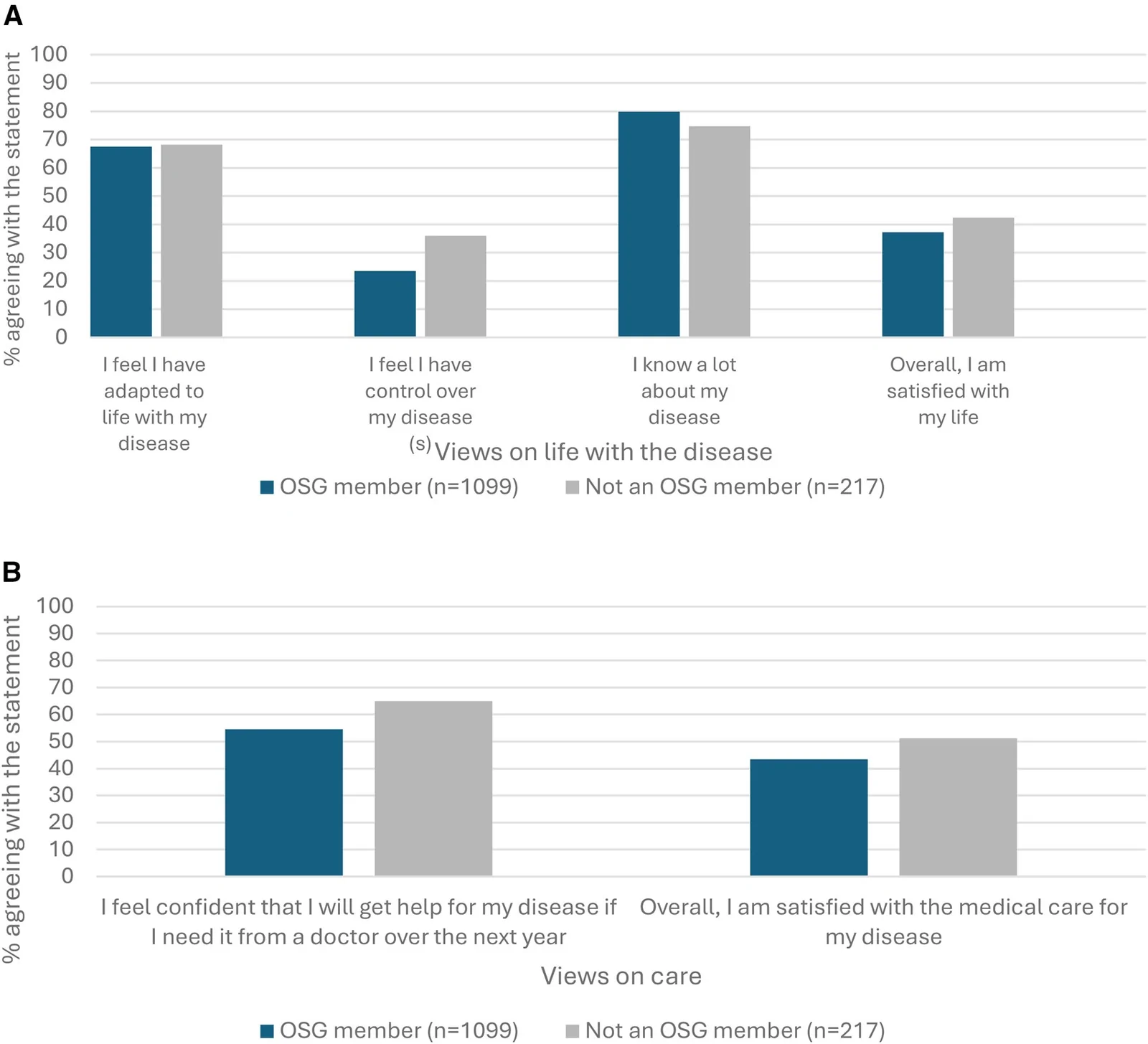
Patient views on life and care. (**A**) Patient views on life with the disease(s). (**B**) Patient views on medical care

Members of OSGs were significantly less satisfied with their medical care (*P* = 0.002) and less confident that they would receive help from a doctor if they needed it over the next year (*P* < 0.001) (Fig. 2B). OSG members also had less trust in GPs and rheumatologists (not significant).

One explanation for this is that patients joined support groups due to traumatic diagnostic journeys, feeling invalidated by clinicians, seeking validation from peers:

‘*Mostly what I see on social media is a lot of positivity, from people supporting and believing each other. Often people are coming to that where they’ve had bad* [experiences in] *medical appointments, but they find support and belief from other patients’* (Neurologist 12).

Additionally, reading others’ negative experiences on OSGs could amplify negative feelings about clinicians. Some clinicians expressed concern and upset about this, especially those named personally in negative posts. Others voiced concerns that negativity was encouraged through the ‘*potentially dangerous culture of these* [online] *groups*’ (Neurologist J).

‘[OSGs are] *generally positive, but some can be quite militant and actually encourage people to not trust their doctors, so mixed I’d say*’ (Psychiatrist 51).

Feeling more supported by other OSG members than clinicians could lead to increased resentment and feeling unsupported by the health services:

*‘I have changed so many things just from the advice given in the group and the impact has been massive to me. I feel they care more than the specialists. Support groups definitely do the work that the NHS/doctors should be doing’* (Female, Sjögren’s).

In our exploratory work, a concern was expressed that patients discussing symptoms and emotions on OSGs may reduce communication with others in their social networks. However, survey data revealed that only 13% stated that OSGs made them talk less to family/friends, and 9% stated that they made them less likely to share symptoms with clinicians (Fig. 3).

**Figure 3:**
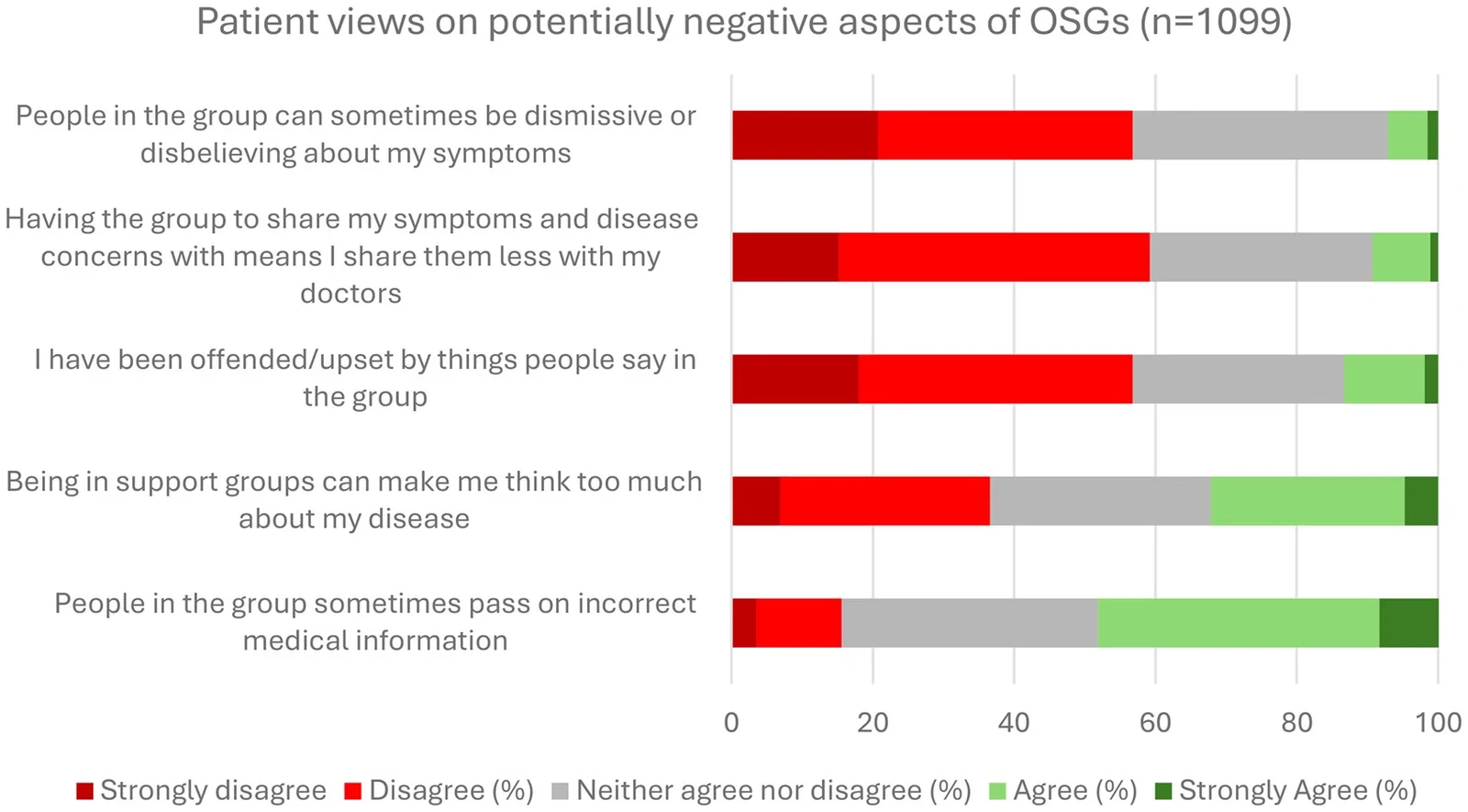
Negative aspects of OSGs

### Theme 3: Negative impacts of OSGs *‘*I tried the forums. I hate the misery.’ (CS0892, RA)

Although many patients reported benefitting from peers’ experience, information from OSGs was reported to blur lines between personal stories and generalizable advice. Both patients and clinicians expressed concerns around how poorly regulated peer support forums could allow the spread of misinformation, particularly around medication and medical authority.

*‘A little knowledge is a dangerous thing as there are too many “experts” in support groups giving advice that they are not qualified to* [give]*’* (CS246, multiple diagnoses).

*‘*[Misinformation and excess concerns about] *hydroxychloroquine is a problem… Visual disturbance, visual loss over time, there seems to a myth around that’* (Rheumatologist Ppt 2).

Clinicians described cases where misinformation from OSGs had caused patients to not heed medical advice:

Participants reported exposure on OSGs to unverified treatments, anti-vaccine discourse or pseudo-medical advice that could influence vulnerable individuals:

‘*Selling of cures… preying on vulnerable and desperate people’* (CS1447, SSc) and ‘*Too much incorrect information, very scary talk about fads, fake news, filled with people believing healthy diet* [conquers] *all. [I] find them frustrating and dangerous*’ (CS1452, multiple diagnoses).

The tone and frequency of these claims varied between online platforms, moderation and group culture.

*‘*[A] *strong moderator makes* [a] *huge difference to quality and ethos of group*’ (CS0254, multiple diagnoses).

This was re-iterated by several clinicians:

‘*Not with LUPUS UK, but if they’ve been doing their own online chatting, no two patients are the same and therefore, so they come in with misinformation or that someone has said something so we tell them…to talk to us and then we can unravel those queries’* (Nurse 20).

Some clinicians were uncomfortable with patients discussing medical matters without a clinician present and advocated for clinician involvement:

‘*The best self-help groups should have clinicians involved’* (Neurologist 41).

However, many of the patient views about the positive impact of OSGs were related to the discussion being open and honest which may be reduced by clinician presence or influence.

Although clinicians expressed various concerns about OSGs in interviews, including the dangers of misinformation, the majority (71%) felt that they were helpful and only 8% felt that they were unhelpful (Fig. 1B). Fig. 2 details patient views of potentially negative aspects of OSGs, with 48% stating that people in OSGs sometimes pass on incorrect information (Fig. 3).

#### Subtheme: Negative peer dynamics

Some participants described that the voices of certain members of OSGs could overpower others and reduce diversity and inclusivity, with one neurologist discussing the potential difficulties of self-selection as self-appointment of OSG leaders (Table 2a).

**Table 2:** Negative experiences of OSGs and groups who can feel marginalized.

	Illustrative quotes
a: Negative experiences	
The most vocal may not be the most suitable or knowledgeable	*‘I think peer support is a double-edged sword, the problem is that many people who put themselves forward for peer support are of a particular ilk, unfortunately those who put themselves forward and are the most vocal are not usually the best people to be providing that peer support’ Ppt 46 neurologist*
Not wanting to be defined by the disease(s)	*CS0081, SLE—‘I don’t want to be defined by my illness. I’m still a productive member of society with a lot to contribute.’*
A constant reminder that you have a disease	*CS0058, SLE—‘There’s a lot of negativity in them and when suffering with depression it doesn’t help you think about other things, there’s a constant reminder of your illness.’*
b: Marginalized groups	
Being younger and having a different ‘mindset’	*CS0032, SLE—‘I feel very out of place being much younger than most group members. I seem to have a very different mindset to the defeated people in these groups.’*
Being male	*CS0722, SLE—‘Totally female centric with non-existent help and support for men with Lupus, I do not feel welcome nor wanted in these groups. And even when I do post in them the chance of anyone responding in a supporting way is almost zero. I am far more likely to be greeted with outright hostility or being ignored.*’
Being from a minoritized ethnic group	*CS0221, SLE—‘you can find so many inquiries and pictures which are* not the same as dark-skinned people.’

*‘Always the same people posting, which can crowd out other people if they are hesitant in coming forward’* (CS0033, RA).

While some patients identified strongly with each other’s experiences, others described forums as less harmonious than they had hoped. Some patients described feeling burdened by ‘toxic positivity’ and the pressure to be optimistic:

*‘A lot of emphasis on being cheerful and exercise. Exercise is impossible for me and I hate being told to be cheerful’* (CS1261, RA).

Others felt alienated by others’ success stories (e.g. running marathons when others were unable to walk due to their disease):

*‘The only thing I really don’t like is when people are relentlessly triumphant’* (CS1345, multiple diagnoses).

Meanwhile, others described ‘toxic negativity’ (many patients):

*‘I hate the misery. Everyone is a “warrior.” I don’t want to be a warrior. I want to have a life and be pain free. I don’t want the disease to be my life. Most people in these groups just want to be in pain and commiserate’* (CS0892, RA).

Quantitively, there were no significant differences in satisfaction with life, adapting self-assessment (Fig. 3) and overall WEMWBS wellbeing scores, between OSG members (42.00) and non-members (42.21).

Other patients reported a competitive atmosphere around suffering:

*‘The one-upmanship of who is the sickest is off-putting’* (CS0005, RA), *and how this could s*ometimes silence or invalidate diverse experiences: *‘I feel* [that] *some people have far worse symptoms than mine and it makes me feel like I shouldn’t moan and I should just get on with it’* (CS0599, UCTD).

While patients can feel encouraged to rant or vent about their symptoms: their posts were sometimes perceived by others as ‘*moaning’* (multiple patients).

*‘The group can be good to offload your feelings to, including depression, and you feel better for getting stuff off your chest that nobody else would understand’* (CS1633, RA).

Some participants felt that focusing on problems could lead to inertia that could detract from people adapting or improving lives as much as possible with the disease(s):

*‘I feel a lot of people use it simply to justify self-pity instead of trying to make things better for themselves*’ (CS0272, RA).

This was re-iterated by a rheumatologist who was discussing the importance of mindset:

‘*You know, some of the stuff that comes out of LUPUS UK is great, but it can also not be helpful at all for some people…I think for some people it just re-enforces that helplessness behaviour, you know’* (Rheumatologist 17).

The newly diagnosed were reported to be particularly vulnerable, as they were in the process of forming expectations about the future of their disease course. Some patients described leaving forums or limiting their exposure due to the constant emphasis on suffering, fear and decline. The repetition of negative experiences could heighten anxiety or reinforce hopelessness, especially during personal health dips:

*‘They can be a bit intense. Sometimes I’m scared by people’s symptoms, and it makes me worry about my own future’* (CS0578, multiple diagnoses).

#### Subtheme: Exclusion and marginalization within peer support

There were reports of exclusion, marginalization and structural levels of inequality within peer support. These indicated that less educated, less confident, male, younger and/or from minoritized ethnicities were more likely to feel ignored or unwelcome:

*‘*[I omit the fact I’m Black because] *you can see they’re predominantly White and I don’t think they would be able* [to understand me]*, they wouldn’t—people have biases, and it’s becoming more, I’m noticing it more because of what’s going on in politics and all around the world people are expressing themselves even more. I just want to be treated as a human being’* (Ppt 9, multiple diagnoses).

Patients from minoritized ethnicities and other under-served groups could also have less access to OSGs*:*

*‘Most of our rheumatic and autoimmune patients are Bangladeshi, so I’m not sure they log in to these lupus society websites in the same way’* (GP 7).

Men were also usually in the minority and some reported feeling isolated, with one expressing:

‘*I couldn’t really relate to* [fellow OSG members] *as a man’* (CS0423, RA).

Further examples of quotes from marginalized groups are in Table 2b.

## Discussion

Overall, OSGs were viewed positively by most participants, echoing existing literature [5, 13]. Patients and clinicians recognized the benefits of OSGs as potentially validating and informative, but voiced concern about misinformation, and erosion of trust in healthcare systems. Quantitative findings reinforced these nuances: although OSG members reported feeling less alone and derived emotional benefit from helping others, they also expressed lower satisfaction with medical care and less perceived control over their illness.

### Validation and patient empowerment

Patients consistently described OSGs as filling an emotional void left by the slow, episodic nature of chronic-illness healthcare. Peer interactions provided existential validation of their symptoms and emotional responses, offering emotional relief unavailable elsewhere. Prior research suggests some specific improvements in physical symptoms as a result of peer support interventions, yet in rheumatology this remains unclear [23]. Nonetheless, these findings support earlier work on the psychology of shared experience as a framework for how peer support can improve patients’ emotional wellbeing and outlook [24]. In our study, validation was particularly valued by patients with rare conditions or invisible symptoms. Dures *et al.* [25] highlight this unmet psychosocial need that OSGs attempt to address.

Wellbeing scores did not differ significantly between OSG members and non-members, despite qualitative reports of emotional improvement, suggesting a complex, possibly non-linear relationship between peer support and health outcomes. This may indicate regression to the mean or suggest that OSGs help those starting from lower baselines to return to parity. Alternatively, self-selection could explain the pattern—individuals with greater distress or lower satisfaction may be more inclined to seek ongoing online support. Longitudinal designs are needed to clarify causality and temporal dynamics [11].

Yet, for many participants, identification with others extended beyond empathy to a form of identity work. Identity-first language—‘lupies’—reflects a shared ownership of disease and the emergence of collective identity. Unlike some cancer support groups, which often accompany a finite, curative trajectory, rheumatology OSGs consistently serve a lifelong, identity-shaping role: support is about living with rather than recovering from (ADAPT framework) [26].

Patients reported joining OSGs to address ongoing informational needs unmet by conventional services that offer infrequent consultations, focused on physical health. This echoes van Uden Kraan’s suggestion that patient-led peer support initiatives arise partly from systemic gaps in care provision for chronic illness [27].

OSGs thus act as decentralized repositories of experiential expertise. Patients pooled information not to ignore medical advice but to gain confidence navigating their disease and the healthcare system. As in Bandura’s model of self-efficacy, this enhanced participants’ sense of agency and capacity to self-advocate [28]. Several patients reported acquiring the language to explain their symptoms and the confidence to request treatments or referrals. However, this empowerment could generate tension: clinicians may feel disempowered or become suspicious of patients who arrived well-informed from online sources. Such dynamics reflect the shifting epistemic boundaries of modern medicine, in which experiential and professional expertise increasingly intersect [29].

Peer support appears therefore not only therapeutic but political; patients reclaim expertise over their bodies and challenge medical hierarchies. OSGs served as incubators of agency and resistance, fostering health literacy and systemic critique [13]. Clinicians sometimes perceiving empowerment as antagonism highlights the need for partnership, not paternalism.

### Challenges

Participants also highlighted challenges within OSGs, including misinformation, emotional overload, erosion of trust and exclusion of those with limited digital literacy. These risks underscore the importance of careful moderation and the need for clinicians to remain engaged with patients’ use of peer support.

Unverified medical ‘expert opinions’ show how unmoderated spaces can blur the boundary between anecdote and evidence. For example, myths about hydroxychloroquine toxicity directly affected treatment adherence. Participants emphasized that effective moderation mitigated these risks, despite inconclusive evidence to support moderator training. Welfare of moderators, often patient volunteers, is a concern [30].

Beyond factual inaccuracy, the emotional tone of OSGs could also be problematic. Patients reported both ‘toxic positivity’ and ‘toxic negativity’, competitive suffering and exclusion of quieter voices. Those experiencing greater symptom burden may be more likely to seek or remain in support groups, making it difficult to determine whether participation improves wellbeing or reflects pre-existing vulnerability. Minority and male participants often felt marginalized, highlighting the need for more inclusive spaces and attention to intersectional factors such as language and digital literacy.

Tension between the benefits and risks of OSGs highlights the ‘void that healthcare cannot fill’: chronic illness care is necessarily infrequent, while emotional and informational needs are continuous.

### Clinical and policy implications

Peer groups may provide support that formal systems cannot. Clinicians might engage constructively by signposting patients to credible, moderated spaces and acknowledging peer support as a legitimate adjunct to care. Charities already perform much of this signposting work; collaboration could enhance evidence-based dissemination. Introducing a trained health-advice nurse or liaison within rheumatology clinics could further bridge gaps between professional and peer information.

Patient-led initiatives should be encouraged, as helping others provides mutual reinforcement of wellbeing [24]. However, targeted efforts are needed to include under-represented groups.

### Limitations and future research

The study’s limitations include selection bias due to recruiting most participants through OSGs, which likely over-represented digitally literate, older, female patients from White, European backgrounds. The cross-sectional design precludes causal inference, and data relied on self-report. Future work should employ longitudinal methods to examine changes in wellbeing and empowerment over time, compare experiences across different rheumatic diseases and other chronic conditions such as cancer or diabetes mellitus and evaluate interventions supporting moderators and peer mentors. Research should also seek to reduce the disparity between OSG members and non-members and draw comparisons between different OSGs.

## Conclusion

OSGs are reshaping the landscape of rheumatology care. They provide vital emotional affirmation, experiential knowledge and a sense of community that formal healthcare often cannot offer. Yet they also introduce challenges of misinformation, exclusion and evolving clinician–patient dynamics. OSGs thus represent both an opportunity and a disruption: they democratize illness knowledge while challenging traditional authority. Harnessing their therapeutic and educational potential through collaboration, inclusivity and appropriate moderation could make peer support a safe and empowering complement to rheumatology care.

## Data Availability

Anonymized data are available on reasonable request to the corresponding author.
